# Longitudinal Relationships Between Social Anxiety, Peer Victimisation, and Perceived Support Among Children

**DOI:** 10.3390/bs16060958

**Published:** 2026-06-10

**Authors:** Ronald M. Rapee, Kay Bussey, Donna Cross, Sally Fitzpatrick

**Affiliations:** 1 Centre for Lifespan Health and Wellbeing, School of Psychological Sciences, Macquarie University, Sydney, NSW 2109, Australia; kay.bussey@mq.edu.au; 2 The Kids Research Institute Australia, Nedlands, WA 6009, Australia; donna.cross@thekids.org.au; 3 School of Population and Global Health, The University of Western Australia, Perth, WA 6009, Australia; 4 Health Research Institute, University of Canberra, Canberra, ACT 2617, Australia; sally.fitzpatrick@canberra.edu.au

**Keywords:** social anxiety, social support, peer victimisation, childhood, longitudinal

## Abstract

Social anxiety, peer victimisation, and social support are key issues during mid-childhood that provide important influence in the later adolescent years. While extensive research has evaluated these constructs in isolation and during adolescence, almost no longitudinal studies have evaluated relationships between them during mid-childhood. The current study obtained self-report measures of social anxiety, peer victimisation, and social support from a close friend, from 7846 students from grades three and four (M age = 9.01 yr), on three occasions over two years. Path analyses examined mediating and moderating relationships, as well as moderation by binary gender. There was minimal moderation by gender on any of the relationships between variables. Over time, there was a stronger positive prediction from social anxiety to later victimisation (*β*’s = 0.09–0.12) than from victimisation to later social anxiety (*β*’s = 0.04–0.07). Social anxiety negatively predicted later social support (*β*’s = −0.05 to −0.09), but social support did not consistently predict later social anxiety (*β*’s = −0.01 to −0.02). Interestingly, the results did not support a buffering effect of social support on peer victimisation; however, these longitudinal data did support several indirect paths. The results highlight the cyclical relationship between social anxiety and peer victimisation but point to social anxiety as a unidirectional predictor of social support from a close friend during the middle to late childhood years. Early intervention for social anxiety may produce positive downstream effects on both peer victimisation and social support.

## 1. Introduction

Peer relationships can be both positive (e.g., social support, social connectedness) and negative (e.g., victimisation, peer rejection) and are central to the development and maintenance of mental disorders (Olweus, 2013; Webb & Zimmer-Gembeck, 2014). The influence of peer relationships and especially their interconnection with mental health is commonly described in adolescence due to the strongly increasing and key role that peers play in adolescent development (Crone & Dahl, 2012). Despite this focus, the salience of peer relationships is not a categorical change but shows a gradual increase across the childhood years. However, the links between peer relationships and mental health are far less commonly studied during childhood.

A common form of mental disorder, social anxiety disorder, can have strong influences on academic functioning, family relationships, and quality of life (de Lijster et al., 2018; Dickson et al., 2024). It is characterised by a core fear of negative evaluation leading to avoidance of a wide range of social situations, including meeting new people, participating in class, being assertive, and joining groups. These characteristics clearly indicate its links with social functioning, and it has been referred to as the prototypical “social-emotional disorder” (Rapee et al., 2019). Social anxiety disorder increases in frequency during the late childhood period, with a mean onset of around 12–13 years of age and is more prevalent in girls than boys (de Lijster et al., 2017; Kessler et al., 2005). Its non-clinical variant, social anxiety, also increases slightly from the end of childhood, and social anxiety scores are also typically higher among girls than boys, although its impact may be greater among boys (Doey et al., 2014). Hence, the mid-childhood period is critically important as a precursor to the peak onset of social anxiety disorder and increased levels of social anxiety.

Social anxiety disorder has been associated with a deficit of positive peer relationships and an excess of negative peer relationships (Ginat-Frolich et al., 2024; Halldorsson & Creswell, 2017). Evidence has indicated that young people high in social anxiety have fewer friends than those with less social anxiety (La Greca & Lopez, 1998) and that this relationship is stronger for girls than boys (La Greca & Lopez, 1998). Furthermore, the quality of these friendships tends to be poorer, and socially anxious youth are more likely to be rejected and less likely to be accepted by their peers (Chiu et al., 2021). While the very nature of social anxiety disorder and its characteristic patterns of avoidance suggest obvious associations with both positive and negative peer relationships, empirical evidence of these relationships, especially during the mid-to-late childhood period, is surprisingly limited. In particular, there are few longitudinal evaluations of the directions of the relationships. Hence, drawing strong conclusions about whether social anxiety drives or is a driver of these relationships is currently not possible.

One key aspect of positive peer relationships, social support, has been consistently positively associated with mental health and life satisfaction among youth (Bi et al., 2021; Tomini et al., 2026; Zhou & Cheng, 2022). Yet considerably less research has evaluated associations specifically with social anxiety. There is a clear distinction between actual social support (i.e., the degree of support that a person can truly count on from their network) and perceived social support (i.e., the amount and quality of support that an individual believes they are able to access) (Yeo et al., 2025). Associations with mental health have been more consistently demonstrated with perceived social support (Lu et al., 2024). Developmentally, social support from close friends takes on particular importance during middle childhood (Xu et al., 2020). During this phase, children are beginning to increase individuation from their primary caregivers, and friendships increase in reciprocity and support. Friendships are also more important than the broader peer relationship for mental health during the middle childhood years (Maunder & Monks, 2019). Because socially withdrawn children demonstrate less social competence than less withdrawn children (Bohlin et al., 2005; Chen et al., 2006), they have friendships that are characterised by less closeness and support (Rubin et al., 2006; Schneider, 1999). Socially anxious children may also receive less support from close friends due to their smaller friendship circles (La Greca & Lopez, 1998). Therefore, socially anxious children may be expected to report less support from close friends. Friendships in middle childhood also appear to be less important for boys than for girls, who are more invested in close friendships and derive greater supportive and emotional benefits (Rose & Rudolph, 2006; Xu et al., 2020). However, the gender discrepancy in friendship quality appears to be smaller in childhood than in adolescence (Rose & Rudolph, 2006). Thus, any relationship between social support and social anxiety may be stronger for girls than for boys, although this discrepancy may be small during the middle childhood years.

Research focusing directly on social anxiety in children and adolescents has shown small to moderate negative associations between social support and social anxiety (Aune et al., 2021; Coyle et al., 2021; La Greca & Lopez, 1998; Pan et al., 2024; Ren & Li, 2020; Yang & Lu, 2022). However, almost all this research has utilised samples of adolescents or mixed child and adolescent ages, meaning that the relationship between social anxiety and perceived social support, specifically among children, is currently unknown. In one exception, a small sample (*N* = 108) of pre-adolescents (although in their first year of high school; M age = 11.8 yr) completed measures of social anxiety and depression at a single time, along with a range of single items assessing diverse aspects of social support (Leeves & Banerjee, 2014). Perceived availability of support from peers and parents were both negatively and similarly correlated with social anxiety. In contrast, among adolescents, research has generally indicated that perceived support from peers shows some of the strongest relationships with mental health (La Greca & Moore, 2005). More specifically, some research has distinguished between support from general class peers and from close friends. Stronger relationships among adolescent samples are typically shown between social anxiety and support from class peers (Coyle & Malecki, 2018; La Greca & Lopez, 1998), although significant negative relationships between social anxiety and perceived support from close friends have also been shown (Coyle & Malecki, 2018). These relationships are also generally smaller for boys than for girls. Given that gender differences in perceived support, especially from close friends, become more apparent from adolescence (Demaray & Malecki, 2002) and that peer relationships in general take on greater salience in adolescence (Crone & Dahl, 2012; Maunder & Monks, 2019), the same associations with social anxiety may not be shown among children and therefore need to be directly evaluated. In sum, it is plausible that during childhood (rather than adolescence), clear relationships between social anxiety and perceived support from a close friend may be shown, and these relationships may be slightly larger in girls or of a similar magnitude in girls and boys. However, these possibilities remain to be evaluated.

There is also evidence that social anxiety in childhood is associated with a greater frequency of negative social interactions, especially peer victimisation. Victimisation by peers refers to repeated aggression or threat of aggression within a relationship that involves a power imbalance, and it can occur via physical aggression, emotional or psychological aggression, or online. Peer victimisation commonly increases in frequency across the primary school years and is most common around grade four (Cross et al., 2025), giving it particular importance in the mid-to-late childhood period. While the total prevalence of victimisation does not differ greatly between girls and boys, there is some evidence for minor differences in form, with boys reporting more experiences of overt victimisation (Jadambaa et al., 2019; Rose & Rudolph, 2006).

A positive, predictive relationship between peer victimisation and social anxiety is consistent with developmental theoretical and empirical accounts and may be predicted in both directions of influence. First, descriptive depictions of children who are the targets of bullying indicate that they are more likely than the average to display unassertive, reticent, and emotive reactions (Guy et al., 2019; Hodges et al., 1999; Juvonen & Graham, 2014). They are also less liked than other children (Guy et al., 2019). These characteristics also typify children high in social anxiety; therefore, it is theoretically likely that social anxiety will predict victimisation. From the reverse perspective, chronic peer victimisation decreases self-confidence and teaches children that the world is a (socially) dangerous place (Chiu et al., 2021). It has also been shown to sensitise children to future social exclusion (Iffland et al., 2014). These attitudes also underpin heightened social anxiety (Leigh et al., 2023). Therefore, it may be predicted that peer victimisation and social anxiety will relate cyclically over time (Pontillo et al., 2019).

Multiple cross-sectional studies support the positive association between physical, relational, and cyber-victimisation and social anxiety among children (Pontillo et al., 2019), yet fewer longitudinal studies exist. The few longitudinal studies generally support the cross-sectional relationships and show that both directions are relevant—peer victimisation predicts social anxiety at a later time, and similarly, social anxiety predicts later peer victimisation (Chiu et al., 2021; Christina et al., 2021; Nicola et al., 2024). Some evidence indicates a stronger relationship between social anxiety and relational victimisation than other forms of victimisation, and a few studies indicate stronger effects with social anxiety than other forms of anxiety (Nicola et al., 2024). Further, peer victimisation appears to be a stronger predictor of social anxiety than other forms of peer interaction (e.g., peer acceptance, social support) (Chiu et al., 2021). The greater perceived emotional impact of peer victimisation among girls than boys (Rose & Rudolph, 2006) suggests that girls might also show a stronger prediction of social anxiety from victimisation. Evidence for this gender-specific relationship is currently sparse.

In addition to their relationships with social anxiety, it is likely that peer victimisation and social support might be directly associated with each other. For example, it makes sense that young people who are frequently victimised will perceive their peers as less supportive and in turn, this may generalise to lowered perceptions of support even from close friends. On the other hand, children who bully are more likely to target children who are socially isolated due to reduced negative reactions from the friendship group (Hodges et al., 1999). It is therefore logical that children with few friends and low social support will be more likely to be victimised. Combining these suggested relationships between social support and peer victimisation with the previously described relationships with social anxiety suggests that mediating relationships with social anxiety are plausible. For example, low social support may predict greater victimisation, which in turn may predict higher social anxiety; peer victimisation may predict lower perceived support, which subsequently predicts greater social anxiety. Of course, the reverse direction (from social anxiety) may also be shown. To date, evidence of mediation among these variables has been evaluated in only one cross-sectional study (Coyle et al., 2021). This study assessed 669 early adolescents in grades six through eight (age not reported) at a single point in time on measures assessing social anxiety, peer victimisation, and perceived social support from a close friend and from peers. Almost all variables were significantly correlated. Models of indirect relationships showed that traditional victimisation predicted perceived support from peers, which predicted social anxiety. In contrast, perceived support from close friends did not provide an indirect path in the relationship between victimisation and social anxiety. However, the relationships showed clear gender differences. Among girls, peer victimisation predicted perceived support from both peers and close friends, which both predicted social anxiety, but the same was not true among boys. While this study pointed to some very interesting relationships, the sample was not large, especially when divided by gender, and the focus was primarily on early adolescence rather than childhood. Most critically, the cross-sectional nature of the data collection did not allow true mediation to be identified.

Theoretically, it has been argued that social support primarily works as a buffer of emotional reactions to negative life events such as peer victimisation (Hodges et al., 1999). Therefore, it is also plausible that social support and peer victimisation will moderate their relationships with social anxiety. Children who have strong support from peers or a close friend may be protected from the negative emotional impacts (including social anxiety) of peer victimisation (Schacter & Juvonen, 2020). The reverse is also possible—social anxiety may predict greater peer victimisation among children who have less social support. Empirical evidence for this buffering hypothesis shows some modest support (Hodges et al., 1999), although effects may be more inconsistent among girls than boys (Desjardins & Leadbeater, 2011). However, evidence on social anxiety is almost non-existent. In one exception, 1700 grade eight students completed measures of social anxiety, peer victimisation, and perceived support from a close friend (Schacter & Juvonen, 2020). Among boys, the relationship between peer victimisation and social anxiety was not significantly moderated by social support from a best friend. In contrast, girls showed a complex picture in which an attenuated relationship between social anxiety and peer victimisation was only associated with best friend support when girls also viewed their friend as not being victimised themselves.

Summarising the extant literature, there is evidence that social anxiety, peer victimisation, and social support are all correlated. To date, most evidence, especially involving social anxiety, has focused on adolescents, and evidence of these relationships during middle childhood is less extensive and may well be different, given the different importance of peer relationships across these two developmental stages. More importantly, most research is based on data from a single time point, making directional conclusions impossible. Only a handful of studies have utilised longitudinal designs, and these have often involved relatively small samples, restricting the power to examine gender differences. Finally, no longitudinal study has evaluated the relationships between all three variables among pre-adolescent children in a single, longitudinal dataset.

The current study aimed to evaluate the longitudinal relationships between social anxiety, peer victimisation, and social support among pre-adolescent children in grades three and four at baseline. Based on the literature described above, it was hypothesised that peer victimisation and social anxiety would positively predict each other at a subsequent time point among both girls and boys. In contrast, we expected negative paths between perceived social support from a close friend and social anxiety to only be significant among girls and, similarly, for paths between social support from a friend and peer victimisation (Desjardins & Leadbeater, 2011; Schacter & Juvonen, 2020), although given the limited extent of the prior literature, this prediction was speculative. We evaluated both mediation and moderation models. Indirect paths among the three variables were expected to show social support mediating the paths (in both directions) between peer victimisation and social anxiety and peer victimisation mediating the paths (in both directions) between social support and social anxiety, at least among girls (Coyle et al., 2021). Again, this prediction was not a strong one given the limited prior literature. Moderation models were expected to show significant relationships between social anxiety and the interaction between peer victimisation and social support.

## 2. Method

### 2.1. Design

Data for the current study were drawn from a large, school-based trial of interventions for bullying and anxiety (Rapee et al., 2020). The larger trial involved random allocation to four intervention conditions, which showed no significant differences. Nonetheless, the intervention condition was included as a covariate in all path analyses (see below). Hence, the current study reflects a longitudinal, three-wave design where measures of key variables were taken annually, over two years (baseline, 12 and 24 months).

### 2.2. Participants

The study collected data from students in grades three or four at baseline from 135 primary schools across two states in Australia: New South Wales (*n* = 100 schools) and Western Australia (*n* = 35 schools). The schools represented a wide range of socioeconomic status (SES) areas and included a wide range of school sizes from rural and urban areas. For the current study, participants were included if they provided responses to all of the main variables on at least two time points. The final sample involved 7846 children, ranging in age from 7.51 to 11.54 years at baseline (M age = 9.01, SD = 0.71 yr), including 4055 girls (51.7%) and 3791 boys (48.3%) (Due to a few schools’ policies, gender was only able to be listed as binary).

### 2.3. Measures

Peer victimisation: The Revised Olweus Bully/Victim Questionnaire (OBVQ: Olweus, 1996) is one of the most widely used measures of self-reported bullying and victimisation. The current analyses used the victimisation measure. This measure consists of a description of bullying followed by five items describing specific aspects of (traditional) victimisation over the “past couple of months” (e.g., “I was called mean names, was made fun of, or teased in a hurtful way”). Each item is answered on a 5-point scale from 1 (has not happened) to 5 (several times per week), and higher scores reflect more frequently experienced victimisation. This shorter version of the OBVQ has not been widely used, but the longer version has shown strong psychometric properties (Olweus, 1996). In the current sample, alphas for the short version were 0.70, 0.68, and 0.68 at the three time points.

Social anxiety: Social anxiety was assessed with the social anxiety subscale of the Spence Children’s Anxiety Scale (SCAS-Soc: Spence, 1998). This 6-item self-report measure assesses standard symptoms of social anxiety (e.g., “I worry what other people think of me”) and is a very widely used measure that shows strong psychometric properties (Spence, 1998). Higher scores indicate more anxiety in social situations. In the current sample, alphas were 0.75, 0.77, and 0.77 at the three time points.

Social support: The Child and Adolescent Social Support Scale (CASSS: Malecki & Demaray, 2002) assesses perceived support from five different sources. Due to both its potential importance during mid-childhood and to questionnaire load limits, the current study used only the subscale reflecting perceived support from close friends, which consists of 12 items (e.g., “My close friend: gives me good advice”), rated on a 6-point scale (1—never to 6—always). Higher scores reflect greater perceived support. The subscale has shown good psychometric properties (Malecki & Demaray, 2002), and in the current sample, alphas were 0.93, 0.94, and 0.95 at the three time points.

### 2.4. Procedure

The larger study, including all measures for the current analyses, was approved primarily by the Macquarie University Human Research Ethics Committee and was subsequently approved by a large range of ethics committees across the relevant partner organisations. Prior to testing, caregivers of participating students provided signed, opt-in consent, following which all students provided informed assent to participate. Testing was completed in school classrooms under teacher supervision. Measures were completed individually, mostly via the online platform, Qualtrics (around 15% of schools administered questionnaires on paper). Testing was repeated approximately 12 and 24 months after baseline in each school.

### 2.5. Data Analysis

Data were initially manipulated with SPSS version 31.0, and the main path analysis was conducted in AMOS version 30.0. Following an examination of missing data, bivariate correlations between the primary study variables, victimisation, social anxiety, and social support, were calculated separately for boys and girls within each time point. The main questions of interest were addressed with two multiple-group path analyses estimating relationships between the primary variables across the three time points, moderated by participant gender and also including intervention condition and age (see missing data below) as covariates. One analysis examined moderation and the other, mediation. Missing data were handled within AMOS using full information maximum likelihood (FIML) estimation. All variables showed acceptable normality, with skew and kurtosis all <2. All outcome variables were first standardised. Stabilities across time were estimated and controlled in the model within each variable, and the relationships within each time point (cross-sectional relationships) were also estimated in the models. No other error terms were permitted to covary. To evaluate the influence of the moderation of victimisation and social support, the standardised estimates of each of these variables were multiplied at each time point. Given that the primary focus of interest with respect to gender was for the relationships between variables, only the structural weights model (where paths between variables were constrained to be equal between girls and boys) was estimated and compared against the unconstrained model. Models were compared by examining the change in chi-square, and the model fit was evaluated according to the comparative fit index (CFI), relative fit index (RFI), and the root mean square error of approximation (RMSEA). Standardised regression weights were interpreted in line with suggestions by Orth et al. (2024) for cross-lagged effects: 0.03—small; 0.07—medium, 0.12—large. To obtain these guidelines, Orth and colleagues reviewed a representative sample of studies that utilised cross-lagged models within four sub-fields of psychology (including developmental and clinical) and calculated the effect sizes corresponding to the 25th, 50th, and 75th percentiles. Hence, these recommended descriptors represent effect sizes relative to effects described in psychological research. For example, an effect of 0.07 sits within the upper half of effects across these fields of psychology. To estimate mediation paths in the mediation model, two new datasets were created using stochastic regression imputation and the 95% confidence intervals were estimated using 5000 bootstrap estimates.

## 3. Results

### 3.1. Missing Data

The full sample included 8796 students, of whom 7846 provided data on two or more occasions and were included in analyses. To determine the generalisability of this sample, participants who were missing data on more than one occasion (950, 10.8%) were compared to the included sample on demographic data and the key variables. Differences between the samples were extremely minor. Participants with missing data were significantly older (M age = 9.58, SD = 0.62 yr) than participants in the analyses (M age = 9.50, SD = 0.61 yr), but the difference was very small, *t*(8431) = −3.54, *p* < 0.001, *d* = −0.13. Nonetheless, given the significant difference, age was included as a covariate in the path analyses. They also reported significantly lower social support with an extremely small effect size (M = 3.31, SD = 1.17 vs. M = 3.38, SD = 1.15), *t*(8244) = 1.77, *p* = 0.039, *d* = 0.07. Mean scores on victimisation, *t*(8419) = −1.32, *p* = 0.093, *d* = −0.05, social anxiety, *t*(8393) = 0.12, *p* = 0.453, *d* < 0.01, gender, χ^2^(N = 8796) = 3.16, *p* = 0.076, and school region, χ^2^(N = 8796) = 0.04, *p* = 0.947, *d* < 0.01, did not differ significantly between the samples. Given these extremely small differences (due to the large sample size), missing data were assumed to be missing at random.

### 3.2. Bivariate Relationships

Pearson bivariate correlations between the three sets of variables at each time point are shown in Table 1. As can be seen, concurrent correlations between victimisation and social anxiety were positive and in the moderate range; between victimisation and social support were negative and slightly smaller; and between social anxiety and social support were negative and mostly small. Correlations were minimally but consistently larger for girls than for boys.

### 3.3. Path Analyses—Moderation of Victimisation and Social Support

**Figure 1:**
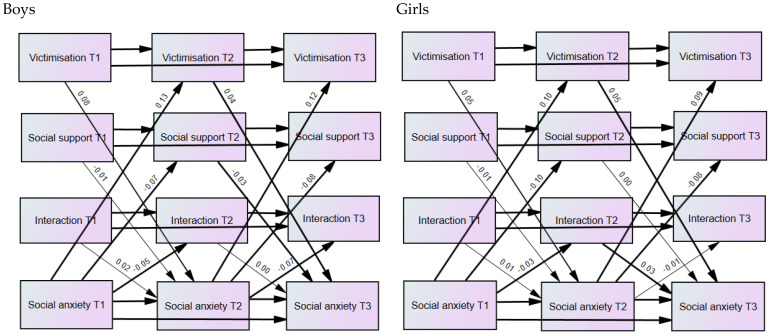
Moderation model indicating standardised paths between outcome variables over time. Separate models are shown for boys and girls (unconstrained). Note: Thinner lines indicate not statistically significant at *p* < 0.05. Paths show standardised regression weights.

**Figure 2:**
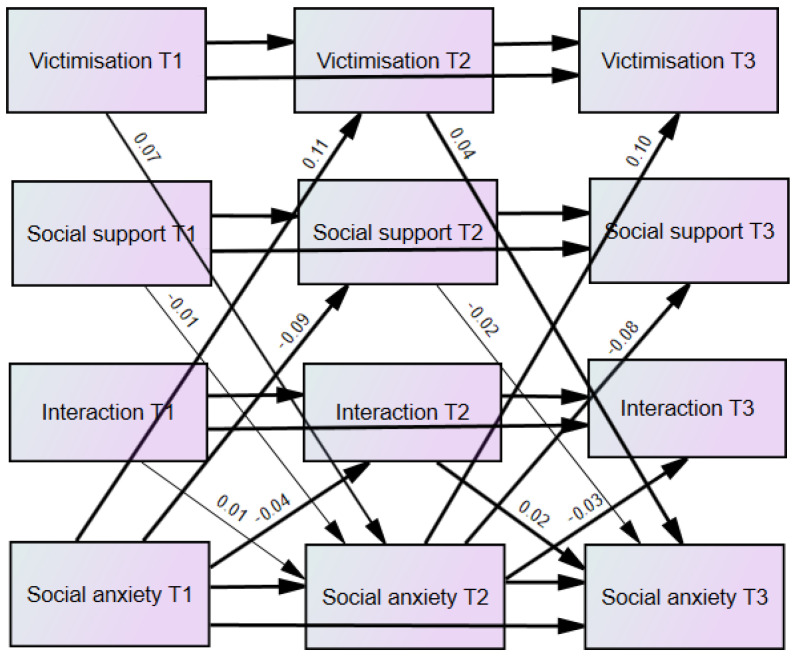
Moderation model indicating standardised paths between outcome variables over time, constrained to be equal between girls and boys. Note: Thinner lines indicate not statistically significant at *p* < 0.05. Paths show standardised regression weights.

The unconstrained model evaluating paths between variables, including the moderation variable between victimisation and support, fit the data well, χ^2^(50) = 249.54, *p* < 0.001, CFI = 0.99; RFI = 0.94; RMSEA = 0.02. The constrained model showed a significantly worse model fit, Δχ^2^(24) = 55.5, *p* < 0.001, indicating statistically significant gender differences. However, the very large sample meant that trivial differences could be significant, and the fit indices showed that the constrained model still fit the data very well with fit indices essentially equivalent to those of the unconstrained model, χ^2^(74) = 305.03, *p* < 0.001, CFI = 0.99; RFI = 0.95; RMSEA = 0.02. Further, examination of the separate paths for girls and boys in the unconstrained model showed only very minor differences (see Figure 1). Hence, despite statistical significance, the constrained model was seen as the most parsimonious fit and is shown in Figure 2.

### 3.4. Path Analyses—Mediated Relationships

**Figure 3:**
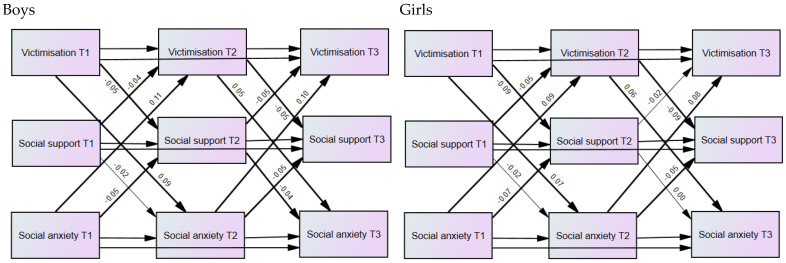
Mediation model indicating standardised paths between outcome variables over time. Separate models are shown for boys and girls (unconstrained). Note: Thinner lines indicate not statistically significant at *p* < 0.05. Paths show standardised regression weights.

**Figure 4:**
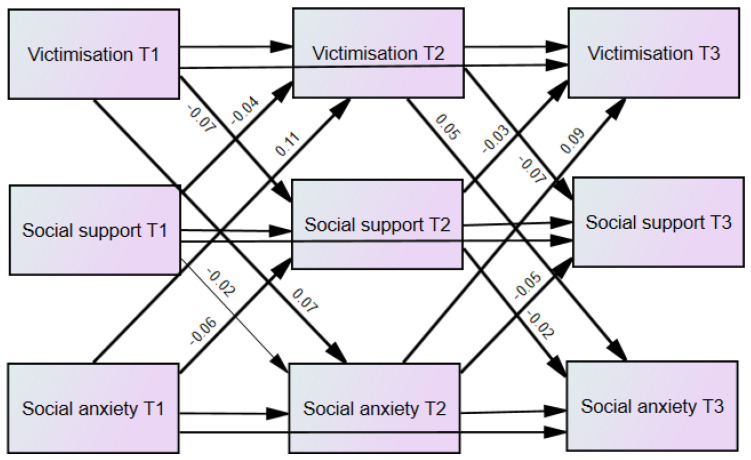
Mediation model indicating standardised paths between outcome variables over time, constrained to be equal between girls and boys. Note: Thinner lines indicate not statistically significant at *p* < 0.05. Paths show standardised regression weights.

The unconstrained model, including paths between victimisation and support, fit the data well, χ^2^(14) = 28.92, *p* = 0.011, CFI = 1.00; RFI = 0.98; RMSEA = 0.01. The constrained model showed a significantly worse model fit, Δχ^2^(21) = 39.57, *p* = 0.008, indicating statistically significant gender differences; however, as above, this model still fit the data very well and the fit indices between the two models showed no obvious differences, χ^2^(35) = 68.48, *p* = 0.001, CFI = 1.00; RFI = 0.98; RMSEA = 0.01. Examination of the separate paths for girls and boys in the unconstrained model showed only very minor differences (see Figure 3). Therefore, the constrained model was accepted as providing the most parsimonious fit and is shown in Figure 4. In addition to the main effects shown in Figure 4, analyses indicated significant indirect paths between T1 and T3 for all three of the primary variables (all *β*’s > 0.03; *p*’s < 0.001).

## 4. Discussion

The current study provided the first longitudinal evaluation of the relationships between peer victimisation, social support from a close friend, and social anxiety in a large sample of children during the middle childhood years. The largest identified main effect relationships were the paths from social anxiety to peer victimisation a year later at each wave (*β*’s = 0.09–0.12) (medium to large effects; Orth et al., 2024). The data also showed consistent prediction in the opposite direction, peer victimisation to social anxiety; however, these relationships were smaller, showing small to medium effect sizes (Orth et al., 2024) (*β*’s = 0.04–0.07). Small to medium bi-directional, negative relationships were shown between peer victimisation and perceived social support, although victimisation predicted support slightly more (*β*’s = −0.07; medium effects) than the reverse (*β*’s = −0.03 to −0.05; small effects). In contrast, significant longitudinal relationships between perceived social support from a close friend and social anxiety were shown in only one direction—social anxiety negatively predicted later support (*β*’s = −0.03 to −0.06; small to medium effects). When extrapolated across the three time points, statistically significant indirect paths were shown between each of the three main variables, including victimisation to social anxiety via social support and social anxiety to victimisation via social support and social support to social anxiety via victimisation, and social anxiety to social support via victimisation. Most of these indirect paths were in the small to medium range. The relationships between social anxiety and the interaction of peer victimisation and perceived social support were very small and mostly not significant. Interestingly, although statistically significant gender differences in the patterns of regression weights were shown, likely due to the very large sample, these differences were extremely small (most differences in *β*’s < 0.03) and fit indices between constrained and unconstrained models were almost identical, suggesting that the most parsimonious model was one that was consistent across girls and boys.

The bi-directional relationships between peer victimisation and social anxiety are consistent with previous research, most of which has been conducted among adolescent samples (Chiu et al., 2021; Christina et al., 2021; Nicola et al., 2024). Our research expands this finding to the period prior to adolescence. Interestingly, our results showed that the strength of this relationship appears to be stronger in the direction from social anxiety to victimisation than the reverse. Hence, it appears that children who are socially anxious may have an increased vulnerability to bullying. This finding is consistent with evidence that highly socially anxious children are viewed by their peers as less likable and more likely to be bullied than children low in social anxiety (Luchetti & Rapee, 2014; Verduin & Kendall, 2008) and are less likely to evoke support from bystanders (Laninga-Wijnen et al., 2025). The bi-directional results also suggest a cascading pattern whereby socially anxious children may be more likely to be bullied, which in turn, may exacerbate their social anxiety.

An even bigger directional difference was shown between social anxiety and social support. Social anxiety negatively predicted later perceived support, while the reverse relationship was considerably smaller and mostly not significant. Some evidence has shown stronger relationships between social anxiety and perceived support from class peers than from close friends among adolescent samples (Coyle & Malecki, 2018). Therefore, it is possible that a different pattern may have emerged if we had assessed perceived support from peers. On the other hand, these slightly different patterns of results may reflect the different developmental stages in these studies. Based on the current results with children, it appears that low perceived support from close friends is more likely to be a consequence of social anxiety than a direct predictor. Hence, it is possible that the effect identified in this study is largely perceptual, consistent with evidence that socially anxious children show biases in the interpretation of ambiguous social information (Calleja & Rapee, 2020; Rapee et al., 2024). Alternatively, it is possible that poorer social performance and interaction skills among socially anxious children (Blöte et al., 2015; Spence et al., 1999) lead to relationships with even their close friends that lack closeness (Rubin et al., 2006; Schneider, 1999). Future research that explores the nature of socially anxious children’s relationships with their close friends, along with the direction of influence, will be valuable to provide a deeper understanding of these processes.

The current study provided minimal support for a buffering hypothesis. The interaction between peer victimisation and social support failed to significantly predict social anxiety; although the reverse direction was statistically significant, it was very small. Some of the literature has shown a buffering effect of social support on the broad mental health effects of peer victimisation, although results were not consistent between genders (Desjardins & Leadbeater, 2011). A considerably smaller portion of the literature has extended this to social anxiety (Schacter & Juvonen, 2020). It should be noted, however, that the study by Schacter and Juvonen (2020) only demonstrated a buffering effect under very limited circumstances—only among girls who also perceived their best friend as not being victimised. Hence, our results are quite consistent with these findings, and it appears from the limited evidence so far that social support from a close friend may not buffer the relationship between peer victimisation and social anxiety in this age group. Of course, it is possible that buffering effects may be found through social support from classmates and the broader peer group, a possibility that requires future research. Far less research has looked at social support as a mediating variable (Coyle et al., 2021), despite strong theoretical reasons to expect both peer victimisation and social anxiety to lead to reduced perceptions of support from friends. Our results extend the cross-sectional research by Coyle et al. (2021) and demonstrate several indirect paths between peer victimisation, social support, and social anxiety.

The lack of any major gender differences ran counter to our expectations. However, developmentally, they make sense. This is one of the few studies in this specific area with a sufficient sample size to confidently evaluate gender differences in these relationships, and it is one of the few studies in this area to focus on middle childhood-aged children. Given the changing roles of peer relationships from childhood to adolescence (Crone & Dahl, 2012), along with increases in gender differences in the importance of relationships with the onset of adolescence (Rapee et al., 2019), the lack of gender differences should be less surprising. It should be noted that our analyses did not evaluate gender differences in the absolute values of each construct. There is little doubt that, even in childhood, girls will report more social anxiety and stronger support from friends than boys. Rather, the time-lagged relationships between the variables do not seem to greatly differ between genders during this developmental stage. In other words, during middle childhood, it appears that social anxiety has a similar relationship with later social support and peer victimisation (and vice versa) among girls and boys.

This study was characterised by an especially large sample, representing a broadly diverse sample of the Australian child population, and measured across three time points. Nevertheless, it had several limitations. Perhaps most critically, due to potential measurement fatigue, we did not include measurement of other sources of social support; hence differences in the patterns of relationships between sources of perceived support cannot be determined. Furthermore, while the use of a longitudinal design is a strength, determining the optimal duration between assessment points is extremely difficult in any study. While it is not clear exactly how quickly the three main variables in this study would affect each other, it is unlikely that 12 months was the optimal time, and this remains a limitation of the current study. Future studies using different time intervals (probably much smaller) would be valuable. Our sampling stopped at the end of the Australian primary schooling system (grade six), and we were therefore unable to evaluate changes in the relationships between variables into adolescence. Additionally, although a strength of our study was the use of widely accepted measures, our measure of peer victimisation showed reliability estimates at the lower end of acceptability. We also did not control for any clustering effects within schools, nor did we utilise a Random Intercept model to allow distinction between within-person and between-person variance. Finally, peer victimisation was assessed entirely by self-report, whereas many authors argue for the importance of reports from external observers (teachers, peers) (Cornell & Cole, 2012; Salmivalli & Peets, 2018). Although self-reports provide arguably the most direct indication of internalising variables such as social anxiety and even victimisation and social support, they introduce the potential for artificial inflation of relationships due to shared method variance, along with the influence of perceptual biases.

The current results point to some interesting findings about the relationships between social anxiety, peer victimisation, and perceived social support during childhood. While causality cannot be determined in a longitudinal design, these directionally informative results point to possible causal influences among these variables that will need to be evaluated in future research. Firstly, they underscore the negative impact of social anxiety on peer relationships during the crucial childhood years (Chiu et al., 2021; Dickson et al., 2024). In turn, these findings underscore the potential social costs of this disorder and ways in which the disorder can maintain or expand over time. More specifically, the indications that social anxiety may negatively impact social support, even within close friendships, support findings that the friendships of socially anxious children are less close, supportive, and beneficial than those of non-anxious children (Rubin et al., 2006; Schneider, 1999), potentially increasing vulnerability to negative experiences and later psychopathology (such as loneliness and depression). In turn, the weaker positive peer relationships appear to increase risk for stronger negative peer relationships (such as victimisation), along with all of its downstream costs. Second, the results point to social variables that may play a role in the development of social anxiety disorder prior to its peak onset in early adolescence (Halldorsson & Creswell, 2017), including peer victimisation and reduced friendship support. This may point to possible targets of value in the development of prevention programmes, especially as children transition into high school. The findings suggest potential value of prevention or early intervention programmes aimed at both the population level (e.g., social–emotional learning to build friendships and school-based programmes to reduce peer victimisation) as well as the individual level (building social skills and reducing social anxiety). Future research might build on these findings to create an evolving picture of the development of social anxiety disorder and peer victimisation, along with the development of comprehensive early interventions.

## Figures and Tables

**Table 1:** Bivariate correlations between pairs of dependent variables.

	BVQ1	BVQ2	BVQ3	SCAS-Soc1	SCAS-Soc2	SCAS-Soc3	CASSS1	CASSS2	CASSS3
BVQ1		0.45	0.37	0.34	0.23	0.22	−0.13	−0.12	−0.11
BVQ2	0.48		0.52	0.25	0.38	0.27	−0.11	−0.21	−0.16
BVQ3	0.37	0.49		0.21	0.30	0.37	−0.10	−0.17	−0.24
SCAS-Soc1	0.41	0.28	0.22		0.46	0.38	−0.12	−0.11	−0.09
SCAS-Soc2	0.26	0.40	0.27	0.47		0.56	−0.08	−0.16	−0.14
SCAS-Soc3	0.21	0.28	0.41	0.40	0.55		−0.10	−0.15	−0.20
CASSS1	−0.16	−0.13	−0.11	−0.13	−0.09	−0.08		0.41	0.32
CASSS2	−0.17	−0.28	−0.17	−0.15	−0.25	−0.15	0.35		0.44
CASSS3	−0.15	−0.21	−0.30	−0.12	−0.17	−0.25	0.28	0.38	

Note: Boys reported above the diagonal; girls below the diagonal. Significance values not reported due to the large sample. BVQ—Olweus Bully Victim Questionnaire; SCAS-Soc—Spence Children’s Anxiety Scale, social anxiety; CASSS—Child and Adolescent Social Support Scale (close friend).

## Data Availability

The data are not publicly available due to the sensitive nature of the sample. However, aspects of the data can be obtained from the first author on request.
